# Significant prognostic value of circulating tumor cells in esophageal cancer patients: A meta-analysis

**DOI:** 10.18632/oncotarget.15012

**Published:** 2017-02-02

**Authors:** Shuyu Wang, Hongyang Du, Guixia Li

**Affiliations:** ^1^ Medical Laboratory Department, Heze Municipal Hospital, Heze, Shandong, 274031 China; ^2^ Heze Centre for Adverse Drug Reactions Monitoring, Heze, Shangdong, 274000, China

**Keywords:** esophageal cancer, circulating tumor cells, meta-analysis

## Abstract

Esophageal cancer is the sixth leading cause of cancer death worldwide. Detection of circulating tumor cells (CTCs) is emerging as a novel strategy for predicting cancer patient prognosis. Here we performed a comprehensive literature search to identify relevant articles in EMbase, PubMed, EBSCO, OVID, Cochrane Database, CNKI, WanFangdata and VIPdata. Meta-analysis was conducted using Stata12.0 software, according to the inclusion and exclusion criteria, extracted data and assessment methodology. Thirteen eligible literature studies were included with a total of 979 esophageal squamous cell carcinoma patients, including 424 CTC-positive and 684 CTC-negative cases. Meta-analysis showed that the presence of CTCs was associated with both worse progression-free/disease-free survival [hazard ration (HR) = 2.32, 95% confidence interval (CI) = 1.57 - 3.43, p < 0.001] and poorer overall survival [HR = 2.64, 95% CI = 1.69 - 4.14, p < 0.001]. Further subgroup analyses demonstrated that CTC-positive patients also showed worse progression-free/disease-free survival and poorer overall survival in different subsets. In summary, our meta-analysis provides strong evidence that detection of CTCs in the peripheral blood is an independent prognostic indicator of poor outcome for esophageal squamous cell carcinoma patients.

## INTRODUCTION

Esophageal cancer is the eighth most frequently diagnosed cancer and the sixth leading cause of cancer death worldwide [1]. Patients with esophageal cancer, compared with those with other cancers, have poorer prognosis because of earlier recurrence and metastasis [2, 3]. Even esophageal cancer patients with no metastasis detectable in the clinic at the time of diagnosis may still die of cancer recurrence after surgery [4, 5]. This suggests that esophageal cancer spreading or metastasis cannot be detected by conventional biochemistry testing, imaging or histopathological methods.

Metastasis involves cancer cell separation from the primary tumor, invasion through the basal membrane into a blood or lymphatic vessel, survival in circulation, extravasation, and colonization of distant metastatic sites. Peripheral blood circulating tumor cells (CTCs) are shed by a primary tumor into vasculature, keep circulating in the blood stream of cancer patients, and are likely to play a critical role in hematogenous metastasis [6]. Clinical implications of CTCs are dependent on the techniques used to isolate them. Each isolation technology has strengths and limitations regarding sensitivity and purity, and may yield different subpopulations of cells [7]. Although detection of CTCs is currently used in many clinical trials, their clinical utility is still under investigation, and a number of issues with regard to CTC detection and characterization remain unclear [8].

In recent years, studies of peripheral blood CTCs suggest that CTCs can be used to predict the progress and prognosis of esophageal cancer [9, 10]. However, current research findings on esophageal CTCs are based on small cohorts of tumor specimen and results are inconsistent. Therefore, in this study, we have performed meta-analysis of worldwide published data to evaluate correlation between esophageal CTCs and patient prognosis.

## RESULTS

### Characteristics of 13 papers on esophageal squamous cell carcinoma CTCs

**Figure 1:**
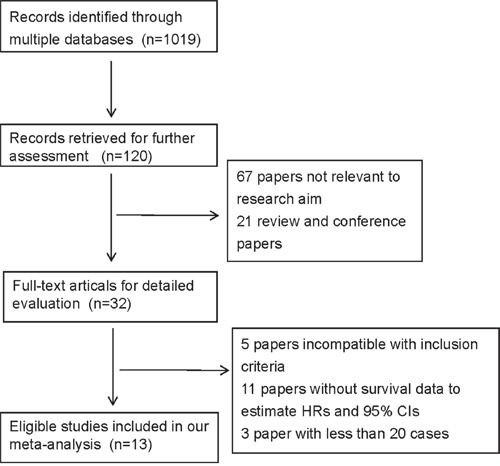
The flow chart of literature search

Literature search was performed to identify articles on esophageal CTCs (Figure 1). A total of 1019 records were initially identified by the comprehensive literature search. After screening the titles and abstracts, 899 irrelevant records were filtered out, and 88 records were subsequently excluded because they were reviews, conference papers, or irrelevant research. There left 32 full-text articles for detailed evaluation, of which 19 studies were further excluded for being incompatible with inclusion criteria [11–15], without survival data to estimate HRs and 95% CIs [1, 16–24, 32] or with less than 20 samples [25–27] (Supplementary Table 1). Finally, 13 eligible studies were included for meta-analysis [9, 10, 28–31, 33–39].

The 13 papers were published between 2006 and 2015, with esophageal squamous cell carcinoma patient sample sizes ranging from 30 to 244 and a total of 979 patients, 424 CTC positive and 684 CTC-negative cases. Method used for CTC detection included RT-PCR (reverse transcription-polymerase chain reaction), CellSearch CTC assay and IE/IF (immunomagnetic enrichment/immunofluorescence staining) approaches. RT-PCR markers for CTCs included carcinoembryonic antigen (CEA) mRNA, Survivin mRNA, squamous cell carcinoma antigen (SCCA) mRNA and cytokeratin (CK19) mRNA. Characteristics of the eligible studies were summarized in Table 1.

**Table 1:** Characteristics of the included studies

Study	Country	Sample size	Sampling time	Median fellow-up (month)	pTNM	Detection method	Detection rate, %(n/N)	Outcomes	HR & 95%CI extraction	HR(95%CI)
Setoyama T (2007)	Japan	125	NR	11.5	I-IV	RT-PCR	61.6(77/125)	DFS	Data extrapolated	0.76(0.55,1.01)
								OS	Data extrapolated	1.03(0.47,2.23)
Cao M(2009)	China	108	Baseline	19.5	I-IV	RT-PCR	47.2(51/108)	PFS	Reported in text	5.18(2.42,8.93)
								OS	Reported in text	5.17(2.30,11.65)
Tanaka K(2010)	Japan	244	Baseline	24.3	I-IV	RT-PCR	8.2 (20/244)	DFS	Data extrapolated	1.96(1.20,3.21)
								OS	Data extrapolated	2.45(1.27,4.72)
		244	Post-therapy	24.3	I-IV	RT-PCR	13.5(33/244)	DFS	Data extrapolated	1.65(1.03,2.63)
								OS	Data extrapolated	1.64(0.91,2.97)
Li J(2012)	China	48	Baseline	34	I-IV	IE/IF	64.6(31/48)	PFS	Data extrapolated	2.44(1.01,5.89)
		48	Post--therapy	34	I-IV	IE/IF	64.6(31/48)	PFS	Data extrapolated	3.89(0.86,17.52)
Yin XD(2012)	China	72	Baseline	24	I-III	RT-PCR	52.7 (34/72)	PFS	Reported in text	3.68(1.37,9.84)
		72	Post--therapy	24	I-III	RT-PCR	30.6 (22/72)	PFS	Reported in text	2.52(0.87,7.23)
Liao HL(2010)	China	62	Baseline	26.4	I-III	RT-PCR	16.1(10/62)	OS	Reported in text	6.53(1.28,6.78)
Matsushita D(2015)	Japan	90	Baseline	10.3	II-IV	CellSearch	27.8(25/90)	OS	Reported in text	2.91(1.44,5.80)
Hoffmann AC(2010)	Germany	25	NR	36	I-IV	RT-PCR	25	OS	Reported in text	10.9( 1.53–77.50)
Honma H(2006)	Japan	46	Baseline	34	I-IV	RT-PCR	30.43(14/46)	PFS	Reported in text	3.00(1.05,8.54)
Yuan X(2012)	China	72	Baseline	24	I-III	RT-PCR	54.2(39/72)	PFS	Reported in text	2.26(0.86,5.86)
		72	Post--therapy	24	I-III	RT-PCR	38.9 (28/72)	PFS	Reported in text	4.08(1.49,11.19)
Wang R(2012)	China	72	Baseline	24	I-III	RT-PCR	44.4(32/72)	PFS	Reported in text	1.94(0.96,3.93)
		72	Post--therapy	24	I-III	RT-PCR	30.6 (22/72)	PFS	Reported in text	2.35(1.16,4.75)
Xue R(2010)	China	57	Baseline	13	I-III	RT-PCR	29.8(17/57)	OS	Data extrapolated	0.55(0.05,5.60)
		57 57	Post—therapy Baseline	13 13	I-III I-III	RT-PCR RT-PCR	3.5 (2/57) 54.4(31/57)	OS OS	Data extrapolated Data extrapolated	3.53(0.16,74.47) 1.39(0.14,13.75)
Guo T(2006)	China	30	Baseline	37	I-IV	RT-PCR	43(13/30)	OS	Data extrapolated	3.53(0.07,166.5)

Abbreviations: RT-PCR, reverse transcription-polymerase chain reaction; IE/IF, immunomagnetic enrichment/immunofluorescence staining; NR, not reported; DFS, disease-free survival; PFS, progression-free survival; OS, overall survival; pTNM, pathological tumour node metastasis. N, sample size; n, the number of positive patients; HR, hazard ratio; CI, confidence interval.

### CTC-positive patients show a higher risk of disease progression and worse overall survival than CTC-negative patients

**Figure 2:**
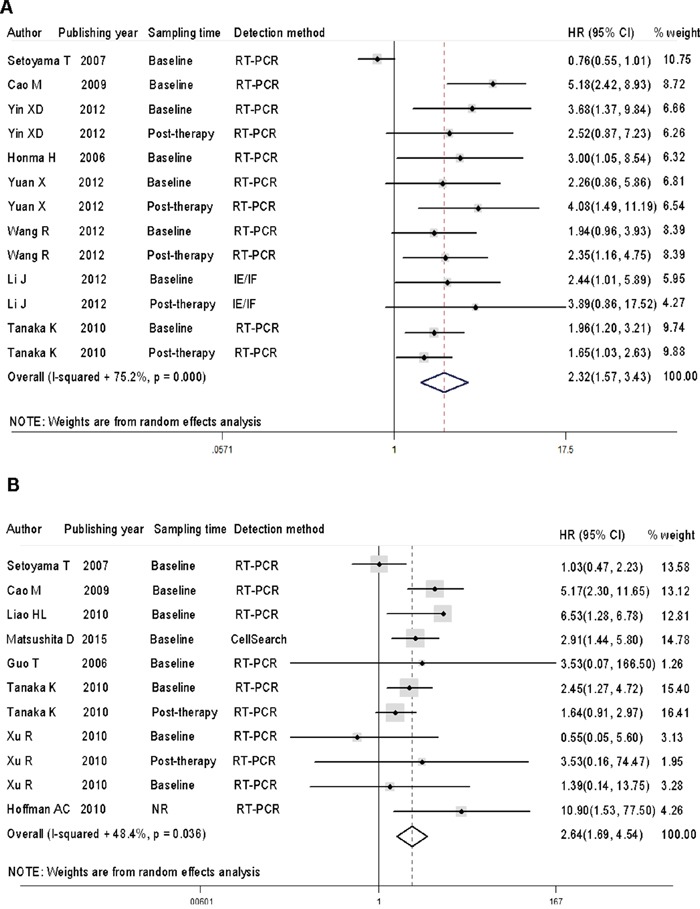
CTC-positive patients show a higher risk of disease progression and worse overall survival than CTC-negative patients **A**. Forest plots of HRs and 95% CIs for disease progression in esophageal cancer patients according to CTC-positive or negative status. **B**. Forest plots of HRs and 95% CIs for overall survival in esophageal cancer patients according to CTC-positive or negative status. NR stood for not reported.

Before performing meta-analysis, heterogeneity tests (or homogeneity tests) were performed using the Cochran Q test and quantitative *I*^2^ test among studies. It was found that both progression-free survival (PFS)/disease-free survival (DFS) (*I*^2^ = 75.2%, p = 0.000) and overall survival (OS) (*I*^2^ = 48.4%, p = 0.036) showed different levels of heterogeneity (Figure 2). So we selected random-effects model for survival analysis.

The HRs for disease progression, measured by PFS and DFS, were available in 8 studies [9, 10, 28–30, 35–37], accounting for 787 esophageal cancer patients. The HRs for OS were available in 5 studies [31, 33, 34, 38, 39], accounting for 264 cases. Analysis of PFS/DFS and OS revealed that, compared with CTC-negative patients, CTC-positive patients had a higher risk of disease progression [HR =2.32, 95% CI = 1.57 – 3.43, p < 0.001] and worse overall survival [HR = 2.64, 95% CI = 1.69 – 4.14, p < 0.001].

### Subgroup analyses reject subgroup heterogeneity and confirm a higher risk of disease progression and worse overall survival in CTC-positive patients

In order to explore sources of heterogeneity and to assess the diagnostic value of CTC status in different subgroups, we performed subgroup analysis (Table 2). In the subgroup analysis based on sampling time, a prognostic effect of CTC detection was confirmed in the analysis of samples collected at baseline (disease progression: HR = 2.65, 95% CI = 1.95-3.60, *P*_heterogeneity_ = 0.334, *I*^2^ = 12.5%; OS: HR = 3.55, 95% CI = 2.46-5.13, *P*_heterogeneity_ = 0.409, *I*^2^ = 1.2%), as well as samples collected post-therapy (disease progression: HR = 2.15, 95% CI = 1.54-3.01, *P*_heterogeneity_ = 0.475, *I*^2^ = 0.0%; OS: HR = 1.64, 95% CI = 0.91-2.96).

**Table 2:** Subgroup analyses of the potential effects of CTCs on survival outcomes in esophageal cancer patients

	Disease progression*				Overall survival			
	n	HR(95%CI)	*p*_heterogeneity_	*I*^2^(%)	n	HR(95%CI)	*p*_heterogeneity_	I^2^(%)
**Sampling time**								
NR	1	0.76(0.56-1.03)	-	-	2	2.80(0.29-27.53)	0.0029	79.2
baseline	7	2.65(1.95-3.60)	0.334	12.5	7	3.39(2.22-5.20)	0.285	19.0
post-therapy	5	2.15(1.54-3.01)	0.475	0	2	1.69(0.94-3.01)	0.631	0
**Sample size**								
<50	3	2.83(1.53-5.24)	0.864	0	5	2.68(0.87-8.27)	0.398	1.5
≧50	10	2.21(1.41-3.47)	<0.001	79.9	6	2.65(1.59-4.43)	0.009	67.3
**Detection method**								
RT-PCR	11	2.26(1.47-3.47)	<0.001	78.4	10	2.61(1.54-4.43)	0.024	53.1
IE/IF	2	2.75(1.28-5.88)	0.601	0				
CellSearch					1	2.81(1.96-4.02)	-	-
**Detection rate(%)**								
<30	2	1.79(1.28-2.51)	0.619	0	5	2.80(1.72-4.54)	0.127	44.3
≧30	11	2.52(1.51-4.20)	<0.001	79.1	5	1.83(0.66-5.07)	0.052	57.3
**CTC markers**								
CEA mRNA	1	0.76(0.56-1.03)	-	-	2	1.06(0.51-2.22)	0.808	0
CK19 mRNA	2	2.14(1.30-3.51)	0.706	0	3	3.06(0.65-14.37)	0.150	47.4
Survivin mRNA	3	4.01(2.49-6.45)	0.375	0	2	5.76(2.72-12.20)	0.491	0
SCCA mRNA	1	3.00(1.05-8.56)	-	-	1	3.53(0.07-172.16)	-	-
CEA & SCCA mRNA	2	1.79(1.28-2.51)	0.619	0	2	1.96(1.27-3.05)	0.373	0
CEA, CK19 & Survivin mRNA	2	3.08(1.50-6.35)	0.606	0	-	-	-	-

* Disease progression outcomes include disease-free survival (DFS) and progression-free survival (PFS).

Abbreviations: RT-PCR, reverse transcription-polymerase chain reaction; IE/IF, Immunomagnetic enrichment/immunofluorescence staining; NR, not reported; CEA, carcinoembryonic antigen; CK19, cytokeratin 19; SCCA, squamous cell carcinoma antigen; CTC, circulating tumor cells; -, when the record number ≦1, the *p*_heterogeneity_ and I^2^ cannot be calculated.

We also explored heterogeneity and the effect of CTC status on outcomes, according to detection methods. CTCs detected by RT-PCR, IE/IF or CellSearch indicated an increased risk for both disease progression (RT-PCR: HR = 2.26, 95% CI = 1.47-3.47, P *P*_heterogeneity_ <0.001, *I*^2^ = 78.4%; IE/IF: HR = 2.75, 95% CI = 1.28-5.88, *P*_heterogeneity_ = 0.601, *I*^2^ = 0.0%; and OS (RT-PCR: HR = 2.61, 95% CI = 1.54-4.43, *P*_heterogeneity_ < 0.024, *I*^2^ = 53.1%; CellSearch: HR = 2.81, 95% CI = 1.96-4.02,) (Table 2).

We next assessed heterogeneity and the effect of CTC status on outcomes, according to CTC markers detected by RT-PCR. All markers except CEA mRNA indicated an increased risk for both disease progression and poor OS (disease progression: HR = 0.76, 95% CI = 0.56-1.03; OS: HR = 1.06, 95% CI = 0.51-2.22, *P*_heterogeneity_ = 0.808, *I*^2^=0.0%) (Table 2). Immportantly, single marker CTC detection was less effective (disease progression for CEA mRNA: 95% CI = 0.56-1.03; OS for CEA mRNA: 95% CI =0.51-2.22; OS for CK19 mRNA: 95% CI = 0.65-14.37; OS for SCCA mRNA: 95% CI = 0.07-172.16), and joint detection using multiple markers improved the effectiveness of CTC detection (disease progression for CEA & SCCA mRNAs: HR = 1.79, 95% CI = 1.28-2.51; DFS for CEA, CK19 & Survivin mRNAs: HR = 3.08, 95% CI = 1.50-6.35; OS for CEA & SCCA mRNAs: HR = 1.96, 95% CI = 1.27-3.05). In addition, the effect of CTC status on patient outcomes was assessed separately for different detection rate and sample size. The result indicated that except OS for post-therapy (95% CI =0.94-3.01) and OS for sample size (<50: 95% CI =0.89-8.27), patients with positive CTCs had a higher risk for poor disease progression than patients with negative CTCs.

### Sensitivity and funnel plot analyses reveal no publication bias in overall and disease progression survival data

**Figure 3:**
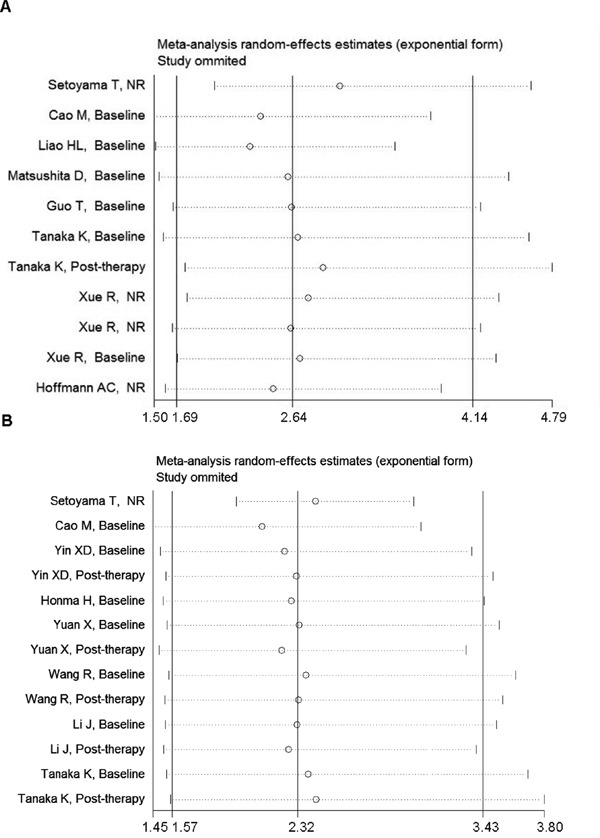
Sensitivity analysis reveals no publication bias in overall and disease progression survival data **A**. Sensitivity analysis of overall survival. **B**. Sensitivity analysis of disease progression survival. NR stood for not reported.

**Figure 4:**
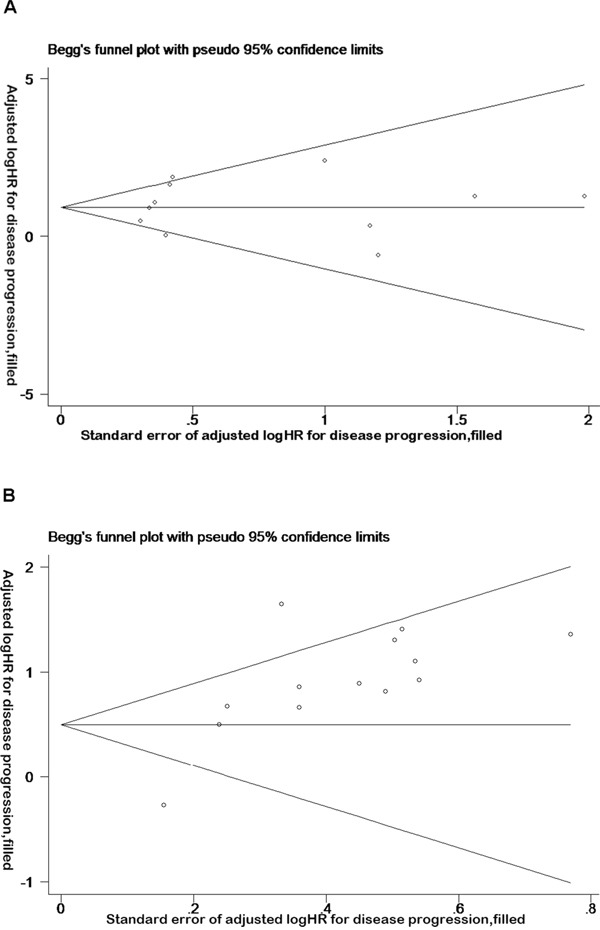
Funnel plot analysis reveals no publication bias in overall and disease progression survival data **A**. Funnel plot of the studies on overall survival. **B**. Funnel plot of the studies on disease progression survival.

Sensitivity analysis was performed on the impact of a single study on the overall results, after studies were removed one by one. It was revealed that no single study can significantly affect the results of the original analysis (Figure 3). In addition, the Begg test indicated that there were no significant publication bias (disease progression: Pr > | z | = 0.077 > 0.05; OS, Pr > | z | = 0.755 > 0.05) (Figure 4), disease progression Egger test, Pr > | z | = 0.001. As the two test results are inconsistent, in order to further examine publication bias, literature disease progression was analysed using the trim and fill method with greater statistical power as below.

### Trim and fill method reveals publication bias in disease progression data

Trim and fill method was developed by Taylor and Tweedie [40]. According to this method, small sample studies were removed and added so as to make the funnel plot symmetric. By removing part of the studies and adding it back, the effects before and after the merger were analysed. If the same conclusion could be drawn, the publication bias was insignificant, and the result was relatively stable [41].

**Figure 5:**
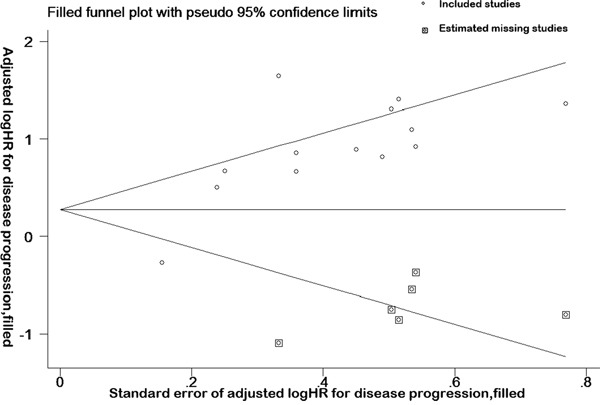
Trim and fill method reveals publication bias in disease progression data Funnel plot and complement funnel plot of the studies on disease progression. The box in this figure is a complement plot, which was used to estimated missing studies.

HRs generated by the fixed effects model and random effects model before trimming were 0.498 (0.322-0.673) and 0.841 (0.448-1.233), indicating that 95% confidence interval was not statistically significant before trimming (Figure 5). However, after adding six points, the HRs were 1.456 (1.239-1.712) and 1.766 (1.256-2.482). The results showed that 95% confidence interval was statistically significantly different after trimming, indicating that the result was unstable. After adding six points to eliminate the impact of publication bias, funnel plot center move to the right (Figure 5). Taken together, the trim and fill method revealed that the publication bias existed in the literature disease progression data.

## DISCUSSION

CTCs are tumor cells released into the peripheral circulation from primary or metastatic tumors spontaneously or due to clinical procedures [42]. Detection of CTCs help monitor tumor recurrence, metastasis and therapeutic responses in real-time, predict patient prognosis and identify mechanisms for tumor progression and metastasis [43]. Recent meta-analyses demonstrate that detection of CTCs in the peripheral blood is an independent prognosticator of poor survival in triple negative breast cancer, ovarian and gastric cancer patients [44–46].

This is the first meta-analysis of CTCs in esophageal squamous cell carcinoma patients, and provide the first comprehensive evidence for CTCs as a biomarker for poor prognosis in esophageal squamous cell carcinoma patients. The results of this study showed that CTC-positive patients had worse prognosis than CTC-negative patients. To explore sources of heterogeneity and the diagnostic value of CTC status, we further performed subgroup analysis, and found that both the baseline and post-therapy positive CTCs were associated with poor prognosis in patients with esophageal cancer. The results suggest that CTCs can be used as a biomarker for poor prognosis in esophageal cancer patients, and that monitoring CTCs at different time points of treatments through repeated CTC testing better predicts patient prognosis.

In this study, subgroup analysis, according to different CTC detection methods, showed poorer prognosis in CTC-positive patients than CTC-negative patients, and showed heterogeneity due to RT-PCR method. To detect the source of heterogeneity in literature CTC studies by RT-PCR, we analysed data from different subsets of RT-PCR markers for CTC detection. Cochrane Q test and *I*^2^ tests of the different CTC markers showed the sources of heterogeneity were eliminated. In addition, our results showed that single marker CTC detection was less effective, and that joint detection using multiple markers can improve the effectiveness of CTC detection in patients in the clinic.

In this study, sensitivity analysis and trim and fill method were also performed. Sensitivity analysis revealed that no single study can significantly affect the results of the original analysis, but the trim and fill method revealed that the publication bias existed in the literature disease progression data.

There were limitations in our meta-analysis. Data collected from studies performed with different experimental approaches by different research groups may lead to significant inconsistency. However, our meta-analysis confirms that detection of CTCs independently correlates with poor prognosis in esophageal cancer patients, irrespective of experimental approaches by different groups. It is therefore safe to conclude that detection of CTCs independently correlates with poor prognosis in esophageal cancer patients.

In summary, this study indicates that CTCs are an important biomarker for esophageal squamous cell carcinoma diagnosis, metastasis and recurrence, and an effective predictor for poor patient prognosis. Detection of CTCs can be used to guide clinical treatments.

## MATERIALS AND METHODS

### Study selection

A comprehensive literature search was performed to identify relevant articles on esophageal CTCs in EMbase, PubMed, EBSCO, OVID, Cochrane Database, CNKI, WanFangdata and VIPdata without any restriction (up to February 2016). The search key words included combinations of carcinoma, cancer, tumor, tumour, circulating tumor cell, circulating tumor cells, circulating tumour cell, circulating tumour cells, CTC, CTCs, esophageal or oesophageal. Studies were considered eligible if they fulfilled all of the following criteria: (1) retrospective or prospective cohort studies; (2) the progression or survival of esophageal cancer patients was stratified and CTC status was verified; (3) hazard ratios (HRs) and 95% confidence intervals (CIs) were provided, or sufficient information was provided for extrapolating them. For studies with overlapping data, we only kept the study with the larger sample size. The study selection process was performed independently by two authors, and any discrepancy was resolved by discussion or consultation with a third party. We did not assign a quality score to each study, because no such score assessment has received a general consensus for non-randomized prognostic studies [45]. Instead, we performed the widely recommended subgroup and sensitivity analyses to determine the potential effects of CTC status on the prognosis of esophageal cancer patients.

### Data collection

Data collected from each eligible study included publication year, author names, country of origin, number of subjects analyzed, esophageal cancer stage, median follow-up day, blood collection time, detection method, detection rate, and cut-off values for CTC status. We also recorded prognostic outcomes including progression-free survival (PFS)/disease-free survival (DFS), overall survival (OS), survival curves, HR and 95% CI, if available, regardless of whether they were tested by multivariate analysis. When more than one blood sample per subject was collected at different time points, each sampling time point was documented and categorized as “baseline” or “mid- or post-therapy”. When more than one method was applied to detect CTCs, all results were considered as independent data sets.

### Statistical methods

The HR and the 95% CI were directly recorded from each study or extrapolated. Survival data were extracted from survival graphs in high-quality PDF documents through Engauge Digitizer 4.1 software, lnHR and SelnHR were calculated, and survival analysis was performed with R programming language [47, 48]. The fixed effect model or random effect model was employed to calculate HR and 95% CI, and statistically significant effect of the combined data was analyzed by the Z value [47, 48]. Heterogeneity between studies was examined using the Cochran Q test and quantitative *I*^2^ test. If p for the Cochran Q test was < 0. 05 or *I*^2^ for the *I*^2^ test was > 50%, study heterogeneity was considered to exist, and random-effects model (DerSimonian Laird) was further used for analysis [49]. If there was no heterogeneity in each study, the fixed effects model (Mantel-Haenszel method) was used for further analysis [50]. Subgroups were identified according to before and after treatments, testing methods and CTC markers, to explore sources of heterogeneity among the groups. Deleting studies one by one allowed sensitivity analysis to assess the impact of a single study on the overall results. Begger funnel plot, Begg test [41] and reduced fill method [51] analyses were used to examine whether there were publication deviations in the published literature, and to assess the authenticity of the original analysis results. Meta analyses were performed using STATA 12. 0 software.

## SUPPLEMENTARY TABLE


