# Supplementary material for: Significant prognostic value of circulating tumor cells in esophageal cancer patients: A meta-analysis
Source: Oncotarget. 2017 Feb 2;8(9):15815–26. doi: 10.18632/oncotarget.15012 (PMC5362525; doi:10.18632/oncotarget.15012)
Supplement: Supplementary file 1 [file oncotarget-08-15815-s001.pdf]

Significant prognostic value of circulating tumor cells in esophageal cancer patients: A meta-analysis

SUPPLEMENTARY TABLE

Supplementary Table 1: The detailed exclusion criteria about the 19 excluded full-text articles

Author, year	Paper titles	Exclusion criteria	References
Li H et al, 2015	Circulating Tumor Cell Analyses in Patients With Esophageal Squamous Cell Carcinoma Using Epithelial Marker-Dependent and -Independent Approaches	No prognostic information	[11]
Ikoma D et al, 2007	Circulating tumor cells and aberrant methylation as tumor markers in patients with esophageal cancer	No prognostic information	[12]
Zhan XK et al, 2008	Clinical analysis of 81 cases with primary small cell carcinoma of the esophagus.	Not involved in circulating tumor cells	[13]
Zhang Y et al, 2011	Expression and clinical significance of PI3K in esophageal squamous cell carcinoma	PI3K is not the marker of circulating tumor cell	[14]
Matsuda S et al, 2014	Clinical significance of plasma fibrinogen level as a predictive marker for postoperative recurrence of esophageal squamous cell carcinoma in patients receiving neoadjuvant treatment	Not involved in circulating tumor cells	[15]
Koike M et al, 2002	Molecular detection of circulating esophageal squamous cell cancer cells in the peripheral blood	without survival data to estimate HRs and 95% CIs	[16]
Hashimoto T et al, 2008	Circulating micrometastases of esophageal cancer detected by carcinoembryonic antigen mRNA reverse transcriptase-polymerase chain reaction: clinical implications	without survival data to estimate HRs and 95% CIs	[17]
Kaganoi J et al, 2004	Detection of circulating oesophageal squamous cancer cells in peripheral blood and its impact on prognosis	without survival data to estimate HRs and 95% CIs	[18]
Brattstrom D et al, 2005	Newly developed assay measuring cytokeratins 8, 18 and 19 in serum is correlated to survival and tumor volume in patients with esophageal carcinoma	without survival data to estimate HRs and 95% CIs	[19]
Ito H et al, 2004	Detection and quantification of circulating tumor cells in patients with esophageal cancer by real-time polymerase chain reaction	without survival data to estimate HRs and 95% CIs	[20]
Nakashima S et al, 2003	Clinical significance of circulating tumor cells in blood by molecular detection and tumor markers in esophageal cancer	without survival data to estimate HRs and 95% CIs	[21]
Geng Q et al, 2010	Detection and quantification of circulating tumor cells in esophageal squamous cancer by real-time fluorescent quantitative reverse transcriptase-polymerase chain reaction	without survival data to estimate HRs and 95% CIs	[22]
Liu Z et al, 2007	Circulating tumor cells in perioperative esophageal cancer patients: quantitative assay system and potential clinical utility	without survival data to estimate HRs and 95% CIs	[23]
Bobek V et al, 2014	Cultivation of circulating tumor cells in esophageal cancer	without survival data to estimate HRs and 95% CIs	[24]
Sclafani F et al, 2014	A pilot study assessing the incidence and clinical significance of circulating tumor cells in esophagogastric cancers	less than 20 samples	[25]
Ren C et al, 2011	Malignant characteristics of circulating tumor cells and corresponding primary tumor in a patient with esophageal squamous cell carcinoma before and after surgery	less than 20 samples	[26]
Fujii K et al, 2010	Primary malignant melanoma of the esophagus--detection of circulating tumor cells	less than 20 samples	[27]
Reeh M et al, 2015	Circulating Tumor Cells as a Biomarker for Preoperative Prognostic Staging in Patients With Esophageal Cancer.	without survival data to estimate HRs and 95% CIs	[32]
Tanaka M et al, 2015	Prognostic significance of circulating tumor cells in patients with advanced esophageal cancer	without survival data to estimate HRs and 95% CIs	[1]
